# Memory Buttons in Combination with Mobile Application-Induced Objective and Subjective Effects in Patients with Atopic Dermatitis

**DOI:** 10.1155/2020/8915893

**Published:** 2020-02-13

**Authors:** Kristina M. Joergensen, Christian Vestergaard, Morten S. Joergensen, Aleksander Eiken, Martin Malmstedt-Miller, Anders N. Ø. Schultz, Mette Deleuran, John R. Zibert

**Affiliations:** ^1^LEO Innovation Lab, Copenhagen, Denmark; ^2^Department of Dermatology, Aarhus University Hospital, Aarhus, Denmark; ^3^Abdominal Center, Department of General Surgery, Herlev Hospital, Copenhagen, Denmark

## Abstract

**Background:**

Atopic dermatitis (AD) is a chronic skin condition where nonadherence often results in lack of disease control.

**Objective:**

We wanted to determine whether the combination of an electronic memory button and a supportive application (app) would affect the Quality of Life and subjective and objective severity measures among AD patients over one month following the patient's normal schedules of treatment.

**Methods:**

A randomized, investigator-blinded, prospective observational feasibility study for one month where patients diagnosed with AD were randomized based on POEM severity score and divided into 3 groups. The 3 groups were (1) the control group with two consultations, (2) in addition to group 1, patients also received electronic memory buttons to click every time they used their topical products, and (3) in addition to group 2, patients also received an app to track their treatment schedules. At both consultations, patients were evaluated using SCORAD, EASI, POEM, and DLQI.

**Results:**

96 patients were enrolled and randomized, of which 83 patients completed the study. EASI and SCORAD scores were lower in all groups at 2^nd^ consultation (*p* < 0.05); however, these were highly significant for group 3 (*p* < 0.05); however, these were highly significant for group 3 (*p* < 0.05); however, these were highly significant for group 3 (*p* < 0.05); however, these were highly significant for group 3 (

**Conclusion:**

A reduction in severity following objective assessments of the AD was observed for all groups and was highly significant for patients offered a memory button and the corresponding app. Furthermore, patients reported a significant subjective beneficial effect if they used the memory button and app. This indicates that digital solutions may have a benefit in clinical practice and may reduce nonadherence and increase the wellbeing of the patients.

## 1. Introduction

Atopic dermatitis (AD) is a chronic skin disease affecting more than 20% of children [1] and 2–18% of adults [2]. Treatment with moisturizers and topical glucocorticosteroids is the first line of treatment, but continuous daily topical administration can be a hurdle to patients resulting in nonadherence [3]. Generally, nonadherence influences the mental and clinical wellbeing of the patient and may result in exacerbation of the disease and comorbidities. It can increase the cost of a treatment regimen and even mortality in rare cases [4–6]. Several studies have successfully tested interventions to improve adherence for eczema patients. These include daily text messages as a reminder aid, workshops, and frequent return visits to clinics [7, 8].

We aimed to determine whether a memory button and an app would affect the Quality of Life (QoL) and subjective and objective severity measures amongst AD patients over one month during the patient's normal schedules of treatment.

## 2. Materials and Methods

The user survey was carried out in accordance with the Helsinki II Declaration and according to the national regulations, which stated it was not considered necessary to report the study to the Scientific Ethics Committee. The purpose of the survey was not to measure safety or effect of the CE-marked device [9]. The data handling of patient sensitive data was approved by the Danish Data Protection Agency and compliant with General Data Protection Regulation.

This randomized, investigator-blinded, prospective observational feasibility study was conducted in Copenhagen and Aarhus, Denmark. The primary endpoints measurements were Dermatology Life Quality Index (DLQI), Patient Oriented Eczema Measure (POEM), SCORing Atopic Dermatitis (SCORAD) and Eczema Area and Severity Index (EASI).

### 2.1. Study Population and Recruitment

All patients were recruited online using advertisements on Facebook and transferred to the website http://www.ades-danmark.dk/ (LEO Innovation Lab, Studies&Me, Copenhagen, Denmark). Patients were initially asked to do a survey of 17 questions including those around demographics, diagnosis, daily treatment, if they own a smartphone/iPhone and contact info. Furthermore, they accepted the study terms and conditions. Patients were invited for a screening visit if they fulfilled the following inclusion criteria: >18 years of age, had received the diagnosis of AD by a general practitioner or a dermatologist, daily use of either moisturizer and/or topical treatment, were able to read Danish, and used an Android phone or an iPhone on a daily basis.

Patients who did not fulfill the inclusion criteria or who were not able to attend in the time period or never answered phone calls were excluded.

Patients were informed verbally and in writing that we, in the process, would follow their treatment; however, they were not informed how the monitoring would be accomplished.

### 2.2. Tools Used during the Study

The SCORAD and the EASI index are validated to record the objective severity of AD [10, 11]. DLQI and POEM [12, 13] are self-assessed measurement tools for monitoring subjective aspects of dermatological disease and AD respectively.

**Figure 1:**
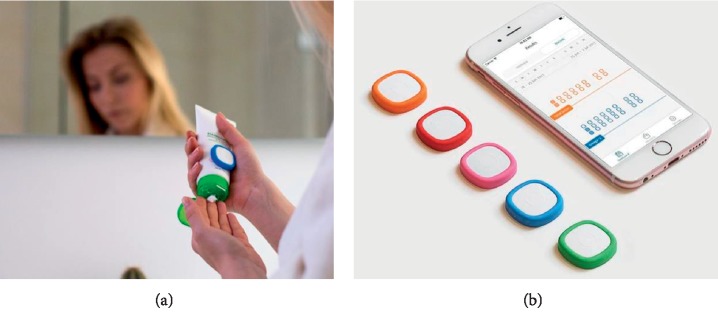
In the study, patients were assigned to one out of three groups, 1: control group, no interference (*n* = 29); (a) 2: intervention group, participants only received the memory buttons without the app (*n* = 34); (b) 3: intervention group, participants received the memory buttons and the app (*n* = 20)).

The memory button is CE-marked [14] (developed by The HabLab Aps, KlikKit, Copenhagen, Denmark) and connected via Bluetooth to a mobile phone and the corresponding app. Both the memory button and the app track the use of the patient's treatment activities when they click the button or signs for usage of treatment on the app, so every click is registered on the app but you can also manually add usage of a specific treatment manually in the app (Figure 1). Furthermore, the memory button also saves the clicks, and they can be transferred to the app later.

### 2.3. Randomization and Interventions

The patients were randomized into three groups based on their POEM score at the first screening consultation in order to distribute the patients equally in groups based on their eczema severity. No intervention was done until the first visit. All patients participated in two consultations with the same doctor, preferably 28 ± 3 days in between. SCORAD, EASI, POEM, and DLQI were assessed at both consultations. Depending on the randomization, they were also introduced to the button and/or app at the first consultation.

The group allocations were: group 1: control group, no interference; group 2: intervention group, participants only received the memory buttons without the app; group 3: intervention group, participants received the memory buttons and the app.

### 2.4. Data Processing and Statistical Analysis

For statistical computing and graphical representations, the R-language was used in Bioconducter (R foundation). Average mean, SD, CI 95%, and *p* value using either ANOVA or Chi^2^ test was used to compare the individual groups. POEM, DLQI, EASI, and SCORAD from first consultation were compared to the second consultation, and for each group, *p* values were calculated using a paired *t*-test to determine if there was a difference between the consultations. As change was evaluated, the data were logarithmically transformed. A significant difference was deemed when *p* < 0.05 and highly significant when *p* < 0.005.

## 3. Results

Nineteen and 99 patients were invited for the first screening consultation in Aarhus and Copenhagen, respectively. Of those, 102 showed up where 5 had absence without notice, and 11 cancelled on the day or prior to the consultation without being interested in booking a new consultation after a reminder email. At the first screening consultation, 6 patients were excluded from the first visit because they did not bring moisturizers for weighing or did not have the symptoms of AD according to Hanifin and Rajka criteria; hence, the total number of included patients was 96. A total of 84 patients also showed up for the second consultation and were included in the final results. All patients were verified by a blinded dermatologist via telemedicine. This resulted in the exclusion of one patient, leaving a total of 83 patients on which the analysis was performed. For all demographic's parameters, no statistically significant differences were found between the three groups, except for the EASI scores where group 2 (*p*=0.028) had more severe eczema than the other groups at baseline (see Table 1).

**Figure 2:**
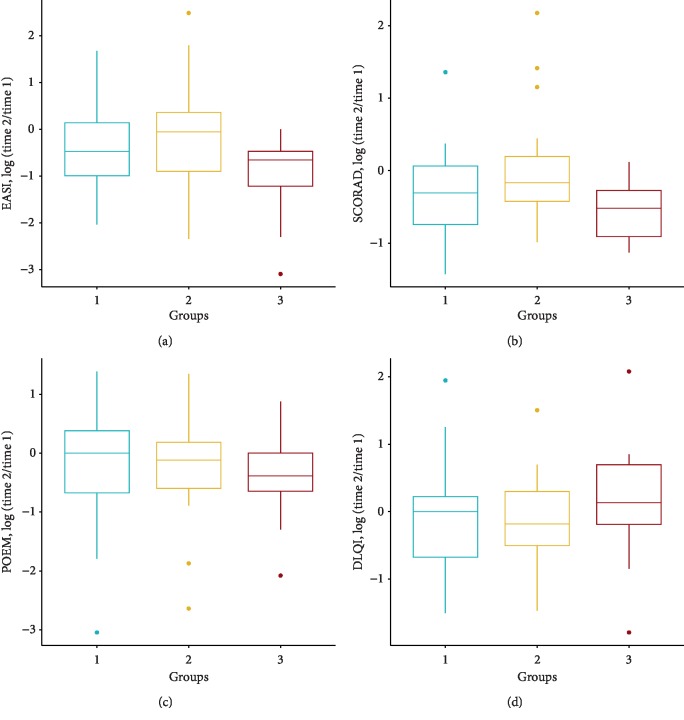
Doctors' evaluation of objective AD severity by (a) EASI and (b) SCORAD. Patients' own assessment of AD severity by (c) POEM (subjective) and (d) perception of Quality of Life (DLQI). Depicted is the amount of change between the two visits (time points) for each of the three groups (1: control group, no interference (*n* = 29); 2: intervention group, participants only received the memory buttons without the app (*n* = 34); 3: intervention group, participants received the memory buttons and the app (*n* = 20)). A significant difference was calculated (*T*-test) between groups 2 and 3 for EASI (*p* < 0.005); for all other comparisons between groups, no significant difference was identified. Between visits for each group, statistically significant change was found for EASI and POEM (all groups), for POEM only in group 3, and no significant change was found for DLQI.

Objective severity measures were statistically significantly decreased in all three groups at the second consultation (Figures 2(a) and 2(b)). Group 3, however, showed a highly significant decrease for both EASI score and SCORAD (*p*=0.0003 and *p*=9.514*e* − 06, respectively). Furthermore, a significant difference was calculated (*T*-test) between groups 2 and 3 for EASI (*p*=0.0062), for all other comparisons between groups no significant difference was identified (Figure 2(a)). For the subjective measurement tools, we found that patients in group 3 had a statistically significant decrease in their POEM score (*p*=0.024), while patients in groups 1 and 2 (Figure 2(c)) did not show any differences. For the measurements of DLQI, we found no statistical significance for difference among the 3 groups from first to second consultation (Figure 2(d)). Absolute values of EASI, SCORAD, POEM, and DLQI at visits 1 and 2 are shown in Table 2.

## 4. Discussion

The study was designed to observe if a memory button and/or app would introduce a behavioral change for patients leading to increased effects of treatment plans interpreted as improved adherence. Patient adherence has, in general, been shown to decrease over time [15] and tend to increase preceding the time of doctor's visits or increase if the frequency of doctor's visits are increased [16].

According to “the health belief model” [17], perceived severity is mediated by perceived threat of a condition and is thus indirectly related to the likelihood of engaging in health-promoting behavior. As observed in our study, including the patient in the treatment process using a memory button and app may therefore promote a behavior change in several ways: (1) giving patients knowledge through tracking and general overview of their treatment could affect the patients' self-efficacy and the likelihood of behavioral change, leading to improved treatment outcomes; (2) the observed lower-perceived severity (POEM) suggest a perceived threat of worsening of their disease symptoms, leading to behavioral change; (3) “a cue to action” phenomenon that directly affects patients and hence lead to a more active treatment behavior.

“The health belief model” has previously been used successfully as a tool itself to create change in health behavior leading to improved adherence [18]. On the other hand, it has also been suggested to result in inconsistent treatment behaviors, leading to lack of effect [19]. However, increased patient involvement and inclusion in the treatment plan by expected use of memory button and app prescribed by a doctor may also support patients in being more satisfied with the treatment result than if they are not able to track their treatment. We did find a clinically objective difference in effect over time within each group and highly significant for the group that received a memory button and app; however, it was clearly observed in patients' subjective measurements of effect when using memory button and app. In a review from 2016, three studies on an app's effect on medication management found that treatment-reminding apps and apps to increase overall adherence did affect patients reported and measured adherence [20]; hence, it can be suggested that the patients' subjective efficacy measures may be interpreted as improved adherence to treatment. Adherence though might or might not drop beyond the 1-month time interval investigated in this survey which is also worth noticing. One way to actually address adherence as an outcome of the memory button and app would be to track actual “clicks” and the use of patients' own products by weighing prior to and after the study; however, this was unfortunately difficult due to lack of a specific treatment plan, patients' own shift of treatments and technical difficulties for the patient.

## 5. Conclusion

In this feasibility study, patients chose their preferred topical treatment and were provided with patient care and education on how to apply medications effectively. This resulted in reduction of severity following objective assessments of the AD and highly significant for patients who were also offered a memory button and corresponding app. Furthermore, patients reported a significant subjective effect if they did use the memory button and app. This indicates that digital solutions may have a place in clinical practice and may reduce nonadherence.

## Figures and Tables

**Table 1:** Patient demographics.

Variables	Group 1 (*n* = 29) M (95% CI)	Group 2 (*n* = 34) M (95% CI)	Group 3 (*n* = 20) M (95% CI)	*p* value
Age	31.1 (26.4–35.8)	32.8 (29.0–36.6)	35.6 (29.0–42.1)	0.505
Sex (%)				0.907^*∗*^
Female	72.4	70.6	70.0	
Male	27.6	29.4	30.0	
Weight	71.5 (65.8–77.2)	71.9 (70.6–73.3)	71.0 (64.8–77.1)	0.970
Height	172.6 (169.2–176.1)	170.5 (165.5–175.4)	174.4 (170.3–178.5)	0.263
BMI	23.9 (22.4–25.4)	24.7 (20.9–28.5)	23.2 (21.6–24.8)	0.411
Asthma (%)				0.994^*∗*^
Yes	31.0	29.4	30.0	
No	69.0	70.6	70.0	
Allergy (%)				0.320^*∗*^
Yes	65.5	79.4	80.0	x
No	34.5	20.6	20.0	
Smoker (%)				0.932^*∗*^
Yes	13.8	11.8	15.0	
No	86.2	88.2	85.0	
Education (%)				0.856^*∗*^
Primary school	6.9	5.9	5.0	
General upper secondary school	27.6	23.5	10.0	
Higher education (less than 3 years)	13.8	11.8	10.0	
Higher education (3–5 years)	27.6	38.2	35.0	
Higher education (more than 5 years)	24.1	20.6	40.0	
No. of medical products	3.1 (2.3–3.8)	3.5 (2.8–4.2)	3.6 (2.2–4.9)	0.645
SCORAD	28.0 (21.5–34.6)	33.3 (26.5–40.2)	27.8 (21.0–34.0)	0.287
EASI	4.7 (2.7–6.8)	10.1 (6.2–14.0)	5.7 (2.7–8.8)	0.028^∧^
DLQI	5.6 (3.7–7.5)	7.0 (5.6–8.4)	5.3 (3.3–7.2)	0.289
POEM	12.0 (9.9–14.1)	13.2 (10.9–15.4)	10.4 (7.1–13.6)	0.283

*p* value calculated with ANOVA if not otherwise specified. ^*∗*^Chi-squared test. ^∧^Below significance level 0.05. 1: control group, no interference; 2: intervention group, participants only received the memory buttons without the app; 3: intervention group, participants received the memory buttons and the app.

**Table 2:** Absolute values of EASI, SCORAD, POEM, and DLQI at visits 1 and 2.

EASI Visit 1	EASI Visit 2	SCORAD Visit 1	SCORAD Visit 2	POEM Visit 1	POEM Visit 2	DLQI Visit 1	DLQI Visit 2
Group 1							
2.6 ± 5.4	2 ± 5.1^*∗*^	26.6 ± 17.3	17.6 ± 12.3^*∗*^	11 ± 5.5	12 ± 5.8	5.0 ± 5.0	6.0 ± 3.2

Group 2							
6.5 ± 11.1	3.7 ± 7.2^*∗*^	32.2 ± 19.6	25.8 ± 14.5^*∗*^	13.0 ± 6.4	10.0 ± 5.9	5.5 ± 4.0	6.5 ± 2.6

Group 3							
2.8 ± 6.4	2.0 ± 5.8^*∗∗*^	32.4 ± 14.3	18.5 ± 13.8^*∗∗*^	13.0 ± 7.1	9.0 ± 6.5^*∗*^	5.0 ± 4.4	6.8 ± 2.5

Within each group, significant change is depicted as follows at visit 2 compared to visit 1. ^*∗*^Significant *p* < 0.05 and ^*∗∗*^highly significant *p* < 0.005.

## Data Availability

The data used to support the findings of this study are available from the corresponding author upon request.
